# NARP-related alterations in the excitatory and inhibitory circuitry of socially isolated mice: developmental insights and implications for autism spectrum disorder

**DOI:** 10.3389/fpsyt.2024.1403476

**Published:** 2024-06-06

**Authors:** Yasunari Yamaguchi, Kazuya Okamura, Kazuhiko Yamamuro, Kazuki Okumura, Takashi Komori, Michihiro Toritsuka, Ryohei Takada, Yosuke Nishihata, Daisuke Ikawa, Takahira Yamauchi, Manabu Makinodan, Hiroki Yoshino, Yasuhiko Saito, Hideo Matsuzaki, Toshifumi Kishimoto, Sohei Kimoto

**Affiliations:** ^1^ Department of Psychiatry, Nara Medical University School of Medicine, Nara, Japan; ^2^ Department of Neuropsychiatry, Wakayama Medical University School of Medicine, Wakayama, Japan; ^3^ Mie Prefectural Mental Medical Center, Mie, Japan; ^4^ Department of Neurophysiology, Nara Medical University School of Medicine, Nara, Japan; ^5^ Research Center for Child Mental Development, University of Fukui, Fukui, Japan

**Keywords:** social isolation, brain development, prefrontal cortex (PFC), neuronal activity-regulated pentraxin (NARP), parvalbumin (PV), social behavior, autism spectrum disorder (ASD)

## Abstract

**Background:**

Social isolation during critical periods of development is associated with alterations in behavior and neuronal circuitry. This study aimed to investigate the immediate and developmental effects of social isolation on firing properties, neuronal activity-regulated pentraxin (NARP) and parvalbumin (PV) expression in the prefrontal cortex (PFC), social behavior in juvenile socially isolated mice, and the biological relevance of NARP expression in autism spectrum disorder (ASD).

**Methods:**

Mice were subjected to social isolation during postnatal days 21–35 (P21–P35) and were compared with group-housed control mice. Firing properties in the PFC pyramidal neurons were altered in P35 socially isolated mice, which might be associated with alterations in NARP and PV expression.

**Results:**

In adulthood, mice that underwent juvenile social isolation exhibited difficulty distinguishing between novel and familiar mice during a social memory task, while maintaining similar levels of social interaction as the control mice. Furthermore, a marked decrease in NARP expression in lymphoblastoid cell lines derived from adolescent humans with ASD as compared to typically developing (TD) humans was found.

**Conclusion:**

Our study highlights the role of electrophysiological properties, as well as NARP and PV expression in the PFC in mediating the developmental consequences of social isolation on behavior.

## 1 Introduction

Neural networks exhibit heightened susceptibility to modulation by cellular activity patterns during the postnatal developmental stage, denoted as the critical period (1). This period, which represents a transient time window for the developing brain, is characterized by the rewiring and consolidation of neuronal networks in response to environmental stimuli (1). Indeed, early life experiences trigger intrinsic mechanisms within a critical period to enhance cortical synaptic plasticity, culminating in enduring large-scale alterations in adult brain systems (2–4). Prior research has demonstrated that aberrant juvenile social encounters, such as neglect or social rejection, exert persistent effects on the structure and function of the prefrontal cortex (PFC), a brain region that plays a central role in higher-level cognitive processes in both rodents (4–8) and humans (9, 10). Notably, these detrimental effects remain resistant to amelioration through subsequent human foster care (9) and rodent resocialization (5). Consequently, the impact of such stress on the PFC circuitry may hold significant relevance during the adolescent phase and could serve as a predisposing element for the emergence or amplification of psychiatric conditions, such as autism spectrum disorder (ASD) and schizophrenia (11–13).

There are two principal neuronal classifications within the cerebral cortex: glutamatergic excitatory pyramidal neurons and GABAergic inhibitory interneurons. Studies utilizing genetically or pharmacologically manipulated mice have primarily focused on the role of inhibitory interneurons during this critical period. In particular, parvalbumin-expressing (PV) neurons, which comprise the most abundant subclass of inhibitory interneurons (14, 15), appear to be intimately involved in critical period plasticity, as PV neurons act as powerful regulators of excitatory pyramidal neuron activity, maintaining an appropriate dynamic range of cortical excitation (16). Furthermore, multiple lines of evidence suggest that the dysfunction of PV neurons within the PFC is implicated in various psychiatric disorders (17–21). Collectively, elucidating the cellular mechanisms driving the developmental plasticity of the prefrontal excitatory and inhibitory circuitry during adolescence is essential for public mental health initiatives and facilitates the development of preventative strategies and novel therapeutic approaches.

We have previously demonstrated that a two-week period of social isolation following weaning induces alterations in the electrophysiological properties of excitatory and inhibitory neurons within the medial PFC of adult mice (7, 8). Furthermore, an immediate influence on specific subtypes of deep-layer excitatory pyramidal and inhibitory neurons at the end of the two-week juvenile social isolation phase was observed (6). However, the extent to which these initial effects on excitatory and inhibitory neurons are accompanied by physiological and molecular changes across the PFC remains poorly understood. Sensory experiences foster the growth and maturation of neuronal circuits, partly by stimulating activity-dependent gene transcription (22). Therefore, neuronal activity-regulated pentraxin (NARP), one of the immediate early gene products, may be one potential molecular factor that affects such alterations following exposure to social isolation during this critical period. NARP is prominently secreted from excitatory pyramidal neurons in an activity-dependent manner and helps in the development of excitatory synapses exclusively on PV neurons (23). Generally, excitatory inputs from pyramidal neurons stimulate PV neurons, which in turn provide feedback inhibition to the pyramidal neurons (24). Consequently, the function of NARP is believed to play a vital role in maintaining cortical excitatory and inhibitory circuitry during critical periods of developmental plasticity (25). Additionally, given the alterations in NARP expression within the PFC in schizophrenia (26), mood disorders (26), and Alzheimer’s disease (27), the NARP gene product may prove integral to the modified properties of excitatory and inhibitory neurons resulting from aberrant juvenile social experiences.

In the present study, using electrophysiological, gene, and protein expression, and behavioral analyses, we explored the timing and molecular processes through which the prefrontal excitatory and inhibitory circuitry is altered in socially isolated mice, with a focus on the expression and function of NARP. Finally, using human blood samples, potential alterations in NARP expression in lymphoblastoid cell line (LCL) samples from patients with ASD were assessed.

## 2 Materials and methods

### 2.1 Mice and housing conditions

C57BL/6J mice were used for all experimental procedures, without duplications between experiments. The animals were housed in our animal facility under standard conditions with a 12-hour light-dark cycle and had libitum access to food and water. Following a previously established protocol (7), a cohort of four male littermates was randomly assigned to either one isolated mouse or three group-housed mice after weaning on postnatal day 21 (P21). The isolated mice were individually housed in cages from P21 to P35 (jSI). GH mice served as the control group for typically developing animals. During the re-socialization period, each isolated mouse was housed with its three littermates until experiments were conducted between P63 and P70. Consequently, the following experiments were conducted to evaluate the effect of social isolation at P35 and/or during adulthood (P63–70). All experiments were approved by the Animal Care and Use Committee of Nara Medical University and conducted according to their guidelines.

### 2.2 Participants

All study participants and their legal guardians provided written informed consent before enrollment. ASD was diagnosed by two experienced child psychiatrists using the criteria outlined in the Diagnostic and Statistical Manual of Mental Disorders 5th edition (DSM-5) and clinical interviews were conducted. The Structured Clinical Interview for DSM-5 was utilized to thoroughly assess any personal or family history of past or present mental illness.

### 2.3 Cell culture

Peripheral blood mononuclear cells (PBMCs) were isolated from venous whole blood collected from 29 TD subjects, averaging 12.0 (SD 2.3) years in age, and from 32 subjects diagnosed with ASD, averaging 11.7 (SD 2.7) years in age (**Supplementary Table 1**). All LCLs were developed by infecting PBMCs with Epstein-Barr virus produced in the supernatant of cultured B95–8 cells, as described previously (28). This study was approved by the Nara Medical University Ethics Committee and was conducted in accordance with the Declaration of Helsinki.

### 2.4 Electrophysiology

Brain slices, including the medial PFC, were prepared from P35 mice (n = 21 cells from 3 mice; GH, n = 24 cells from 3 mice; jSI). The brain was quickly removed under anesthesia with isoflurane and immersed in an ice-cold sucrose-based solution bubbled with a mixed gas of 95% O_2_/5% CO_2_ containing (in mM) 230 sucrose, 2.5 KCl, 25 NaHCO_3_, 1.25 NaH_2_PO_4_, 0.5 CaCl_2_, 10 MgSO_4_, and 10 D-glucose. The frontal cortex was sectioned into 300–330 μm-thick slices in the coronal plane by a vibrating tissue slicer (Linear Slicer Pro 7, Dosaka). Slices were incubated for at least 60 min in a chamber filled with a standard artificial cerebrospinal fluid (ACSF) continuously bubbled with mixed gas, containing (in mM) 125 NaCl, 2.5 KCl, 25 NaHCO_3_, 1.25 NaH_2_PO_4_, 2 CaCl_2_, 1 MgCl_2_, and 25 D-glucose at 32°C, and then maintained in the ACSF at 25°C. Pyramidal cells in the medial PFC layer 3 were current-clamped in the conventional whole-cell configuration using a Multiclamp 700A amplifier (Axon Instruments). Patch pipettes were pulled from the borosilicate glass and filled with an intracellular solution containing (in mM) 141 K-gluconate, 4 KCl, 2 MgCl_2_, 2 Mg-ATP, 0.3 Na-GTP, 0.2 EGTA, 10 HEPES, pH adjusted to 7.25 with KOH. All membrane potentials were corrected for a 13 mV liquid junction potential, as described previously (29). Data acquisition and stimulation were controlled using Signal 4 software with Power 1401 interface equipment (Cambridge Electronics Design).

For the current-clamp recordings, the series resistance was monitored and compensated using a bridge circuit, and the pipette capacitance was compensated. The voltage signals were low-pass filtered at 10 kHz and digitized at 20 kHz. The baseline membrane potential was maintained near -70 mV with current injection. To examine the action potential and subthreshold membrane properties, we recorded the membrane potential responses to depolarizing current pulses from 10–100 pA in 10 pA increments (500-ms duration). The spike threshold was calculated as the voltage at which the slope of the action potential trace reached 10 mV/ms at the rheobase, defined as the minimum current value that elicited at least one action potential. Spike amplitude and spike frequency were defined as the voltage and frequencies of the spikes in response to depolarizing 100 pA current injections from baseline, respectively.

### 2.5 Quantitative PCR

Mouse brains were quickly removed from the skull, and medial PFC sections (Bregma +2.8 mm to +2.1 mm) were microdissected for sample preparations (n = 7; each group at P35, and n = 7; each group at adulthood). From samples of mouse tissues and human LCLs, total RNA was isolated from TRIzol homogenates and purified using the Direct-Zol RNA Miniprep kit (Zymo Research, Irvine, CA, USA) according to the manufacturer’s instructions. First-strand cDNA was synthesized using an iScript kit (Bio-Rad Laboratories, Hercules, CA, USA), and qPCR was performed using SYBR Premix Ex Taq II (Tli RNase H Plus, TAKARA BIO Inc., Otsu, Shiga, Japan) in a StepOne Plus real-time PCR system (Life Technologies, Carlsbad, CA). The specificity of amplification was confirmed by monitoring the dissociation curve at the end of each run. All primer sets (**Supplementary Table 2**) had amplification efficiency ≥ 96% confirmed by standard curve method. Normalization and relative quantification of the expression levels of the target genes were determined by the ΔCT method, using the constitutively expressed genes β-actin (ACTB), glyceraldehyde-3-phosphate dehydrogenase (GAPDH), and cyclophilin (Cyclo) in rodent brains and human LCLs (**Supplementary Table 2**). Furthermore, the levels of these three transcripts in the medial PFC were not affected by age or housing conditions.

### 2.6 Western blotting

The medial PFC sections (Bregma +2.8 mm to +2.1 mm) from mice (n = 7; each group at P35, and n = 7; each group at adulthood) were microdissected, and total protein was immediately isolated using ice-cold homogenizing buffer [50 mM Tris-HCl buffer (pH 7.5), 8 M urea, 0.1 M NaCl, 2 mM EDTA, 1 mM dithiothreitol (DTT)] with cOmplete™ protease inhibitor cocktail (Roche, Mannheim, Germany). The total protein concentration was determined in triplicate using a BCA assay kit (Thermo Fisher Scientific, Rockford, IL, USA), according to the manufacturer’s instructions. Western blotting was performed with the Simple Western™ system, employing capillary electrophoresis to automatically detect and quantify a protein of interest (ProteinSimple, San Jose, CA). In brief, according to the manufacturer’s instructions (ProteinSimple, San Jose, CA), lysates of medial PFC sections (1.5 mg/mL), primary antibodies to Cofilin (#5175; Cell Signaling Technology), NPTX2 (10889–1-AP; Proteintech), and PV (AF5058; R&D Systems) with secondary antibodies were loaded into either 12–230 kDa or 2–20 kDa Separation Module (ProteinSimple) depending on the protein’s molecular weight to run on the Simple Western machine, Wes™ (ProteinSimple). The digital image was analyzed using Compass software, where the quantified data of the detected protein were reported as molecular weight and signal/peak intensity. The expression level of each protein was calculated based on its abundance relative to that of cofilin.

### 2.7 Behavioral analysis in mice

The sociability and social recognition memory of test subjects were measured using a three-chamber test (n = 11; GH, n = 11; jSI). The apparatus consisted of an acrylic open-topped box (60 cm L × 40 cm W × 40 cm H) partitioned into three chambers with two acrylic walls. Each 10-minute phase was initiated by allowing the test mice to freely explore the 3-chamber apparatus for habituation. During the sociability phase, the test mouse was directed to the center chamber, while a wired cup containing a strange (stranger 1) mouse and an empty cup was introduced into the other two chambers. Subsequently, during the social recognition phase, another strange mouse (stranger 2) was introduced into the empty cup, and the movement of the subject mouse was tracked for another 10 min. To counterbalance any possible bias, the orientation of the wired cups containing strangers 1 or 2 (empty) was randomized for each set of experiments. The time spent in each chamber and the time spent exploring the enclosed novel mice or empty cups (novel objects) were recorded using an overhead-mounted camera and analyzed using an automated tracking program (TopScan, Clever Sys Inc.) during the first 4 min of each session (30), with comprehensive data encompassing the entire 10-minute sessions provided in the **Supplementary Figure 1**. To minimize individual differences, the sociability index was calculated as (time spent exploring the stranger 1)/(time spent exploring the stranger 1 + time spent exploring the empty cup), while the social recognition index was calculated as (time spent exploring the stranger 2)/(time spent exploring the stranger 2 + time spent exploring the familiar).

### 2.8 Data analyses

Statistical analyses were performed using the software Prism 8.20 (GraphPad Software Inc., CA, USA). For qPCR analyses, statistical significance was determined using two-way analysis of variance (ANOVA), followed by Tukey’s test. Otherwise, the Student’s t-test or Welch’s t-test was applied based on the outcomes of a test for the equality of variances. All data are presented as mean ± standard error of the mean (SEM), and differences between group means were considered significant if p values showed less than 0.05.

## 3 Results

### 3.1 The excitability of medial PFC pyramidal cells changes after a period of juvenile social isolation

**Figure 1:**
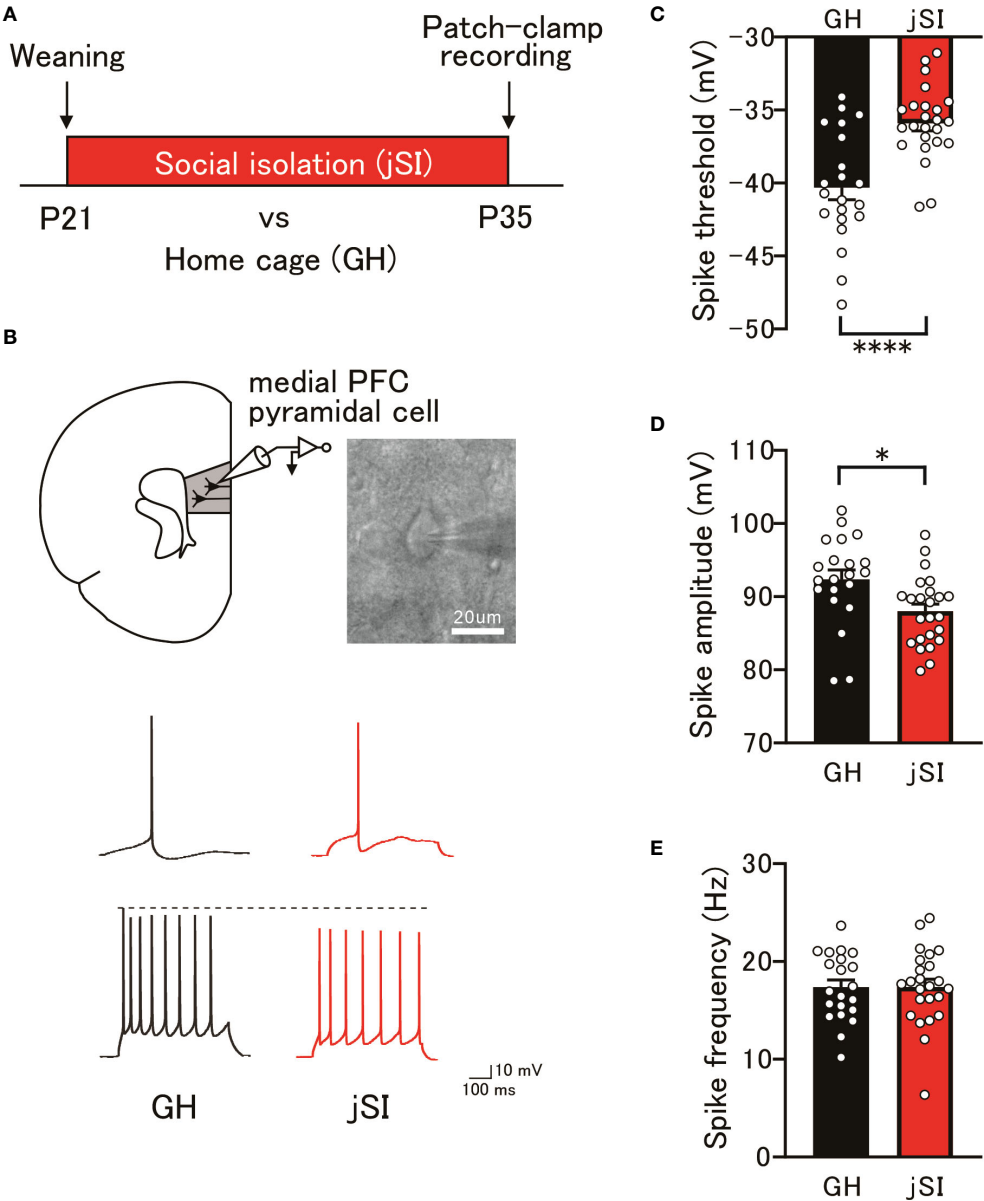
Changes in the excitability of medial PFC pyramidal cells following juvenile social isolation. **(A)** Schematic diagram illustrating juvenile social isolation. Mice underwent social isolation between P21 and P35, with whole-cell patch-clamp recordings performed at P35. **(B)** Top: Current-clamp recordings from pyramidal neurons in the medial PFC layer 3 conducted at P35. Bottom: Representative action potential traces from GH and jSI mice. The top traces depict a spike at the rheobase, while the bottom traces show a spike with a 100 pA current injection. **(C)** jSI mice exhibited significantly higher spike thresholds compared to GH mice [two-tailed *t*-test, t_43_ = 4.571, p < 0.0001, n = 21 (GH), n = 24 (jSI)]. **(D)** The spike amplitude in jSI mice was significantly smaller than in GH mice [two-tailed *t*-test, t_43_ = 2.680, p = 0.0104, n = 21 (GH), n = 24 (jSI)]. **(E)** There was no significant difference in spike frequency between GH and jSI mice [two-tailed *t*-test, t_43_ = 0.03442, p = 0.9727, n = 21 (GH), n = 24 (jSI)]. PFC, Prefrontal Cortex; jSI, Juvenile Social Isolation; GH, Group-Housed; P21, Postnatal Day 21; P35, Postnatal Day 35. * 0.01 ≤ p < 0.05; **** p < 0.0001.

We previously reported that juvenile social isolation (jSI) from postnatal day 21 to 35 (P21–P35) induced long-lasting working memory deficits and altered the electrophysiological properties of excitatory and inhibitory neurons in the adult medial PFC (5, 7, 8). Therefore, we first explored the functional abnormalities that arise after exposure to social isolation (P35) in layer 3 pyramidal neurons, which are considered crucial neuronal substrates for working memory. As shown in **Figures 1A, B**, whole-cell current-clamp recordings were performed on medial PFC pyramidal cells in jSI mice at P35. jSI mice had significantly higher spike thresholds than group-housed (GH) mice of the same age (**Figure 1C**; p < 0.0001). Furthermore, the spike amplitude in jSI mice was significantly smaller than that in GH mice (**Figure 1D**; p = 0.010), although the spike frequency in excitatory pyramidal cells did not differ between the two groups (**Figure 1E**; p = 0.973). Recently, we uncovered reduced excitability of a specific subtype of pyramidal neurons in the deep layer of the medial PFC after jSI at P35 (6). Considering the differences in properties and connections to multiple intracortical and subcortical targets between pyramidal neurons in layers 3 and 5, electrophysiological alterations in medial PFC excitatory neurons might be one of the crucial contributors to the subsequent dysfunction of the entire medial PFC after exposure to social isolation.

### 3.2 Altered expression of NARP may influence the expression of parvalbumin in socially isolated mice

**Figure 2:**
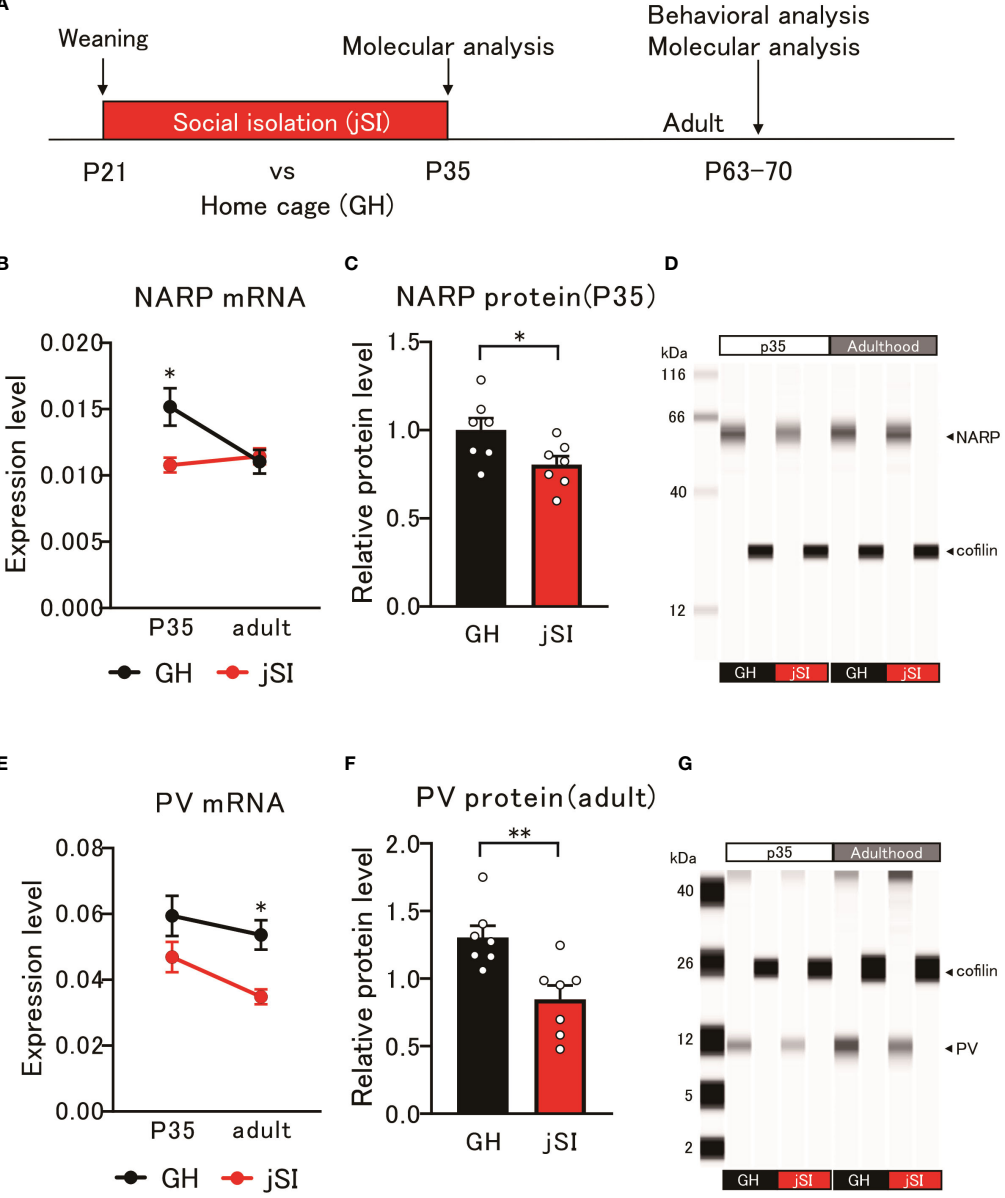
Altered NARP expression impacts the expression of parvalbumin in inhibitory neurons in socially isolated mice. **(A)** Schematic diagram of molecular and behavioral analyses. Molecular analysis was conducted at P35 and adulthood, and behavioral analysis was conducted in adulthood. **(B)** NARP mRNA levels in the medial PFC of jSI mice were lower than those in GH mice at P35, but not at adult [2-way ANOVA, age (P35 and adult) x housing (GH and jSI) interaction F_(1. 24)_ = 6.677 p = 0.0163, age F_(1. 24)_ = 3.505 p = 0.0734, housing F_(1. 24)_ = 4.600 p = 0.0423; Tukey test p = 0.0135 (GH vs jSI at P35), n = 7 (GH, P35), n = 7 (jSI, P35), n = 7 (GH, adult), n = 7 (jSI, adult)]. **(C)** NARP protein levels in the medial PFC of jSI mice were lower than those in GH mice at P35 (two-tailed *t*-test, t_12_ = 2.348, p = 0.0369). **(D)** Representative images of NARP protein expression in the medial PFC of GH and jSI mice. **(E)** PV mRNA levels in the medial PFC of jSI mice were lower than those in GH mice at adult, but not at P35 [2-way ANOVA, age (P35 and adult) x housing (GH and jSI) interaction F_(1. 24)_ = 0.4791, p = 0.4955; age F_(1. 24)_ = 3.821, p = 0.0624; housing F_(1. 24)_ = 11.71, p = 0.0022; Tukey test p = 0.0361 (GH vs jSI at adult), n = 7 (GH, P35), n = 7 (jSI, P35), n = 7 (GH, adult), n = 7 (jSI, adult)]. **(F)** PV protein levels in the medial PFC of jSI mice were lower than those in GH mice at adult (two-tailed *t*-test, t_12_ = 3.440, p = 0.0049). **(G)** Representative images of PV protein expression in the medial PFC of GH and jSI mice. NARP, Neuronal Activity-Regulated Pentraxin; PV, Parvalbumin; PFC, Prefrontal Cortex; GH, Group-Housed; jSI, Juvenile Social Isolation; P35, Postnatal Day 35; Adult/Adulthood, Postnatal Day 63–70; ANOVA, Analysis of Variance. * 0.01 ≤ p < 0.05; ** 0.001 ≤ p < 0.01.

As previously mentioned, NARP secreted from pyramidal cells influences the activity of PV neurons, which are required for homeostatic synapse scaling (22). Considering the dampened firing properties observed in both layer 3 (**Figure 1**) and the specific subtype of layer 5 pyramidal neurons (6–8) within the medial PFC after jSI, we hypothesized that jSI influences the expressions of NARP and PV in the medial PFC during brain development (**Figure 2A**). Using quantitative PCR (qPCR), we found that the mRNA levels of NARP and PV in the medial PFC exhibited distinct expression patterns in jSI mice compared to GH mice (**Figures 2B, E**). Indeed, the developmental trajectories of NARP and PV mRNA expression in GH mice aligned with trends in prior studies (26, 31–33). However, NARP mRNA levels were significantly influenced by housing condition (2-way ANOVA: p = 0.04) and interaction effects (2-way ANOVA: p = 0.016), with a trend effect for age (2-way ANOVA: p = 0.07). Tukey’s *post-hoc* analyses revealed that jSI mice at P35 had lower NARP mRNA levels than GH mice of the same age (p = 0.013). In contrast, PV mRNA levels were significantly influenced by housing conditions (2-way ANOVA: p = 0.002) and showed a trend with age (2-way ANOVA: p = 0.06). Tukey’s *post-hoc* analyses showed that jSI mice had lower PV mRNA levels than GH mice in adulthood (p = 0.036). Western blot analysis confirmed that the protein levels of NARP, but not PV, were significantly lower at P35 in jSI mice than in GH mice (**Figures 2C, D**, p = 0.037). Moreover, the protein levels of PV, but not NARP, were significantly lower in jSI mice than in GH mice during adulthood (**Figures 2F, G**, p = 0.005). These data suggest that relatively lower levels of NARP during the critical period may subsequently affect PV expression in the adult medial PFC, which may contribute to the altered electrophysiological properties of prefrontal excitatory and inhibitory neurons in adult jSI mice.

### 3.3 Social isolation during the critical period affects behavior in adult mice

**Figure 3:**
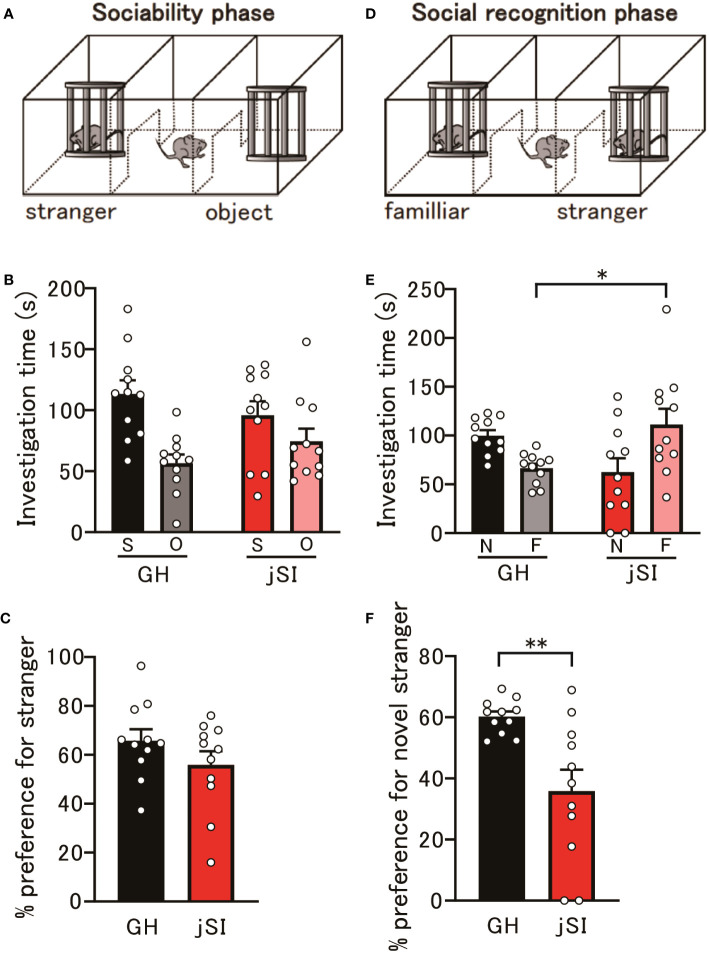
Social isolation during the critical period affects behavior in adult mice. **(A)** Schematic diagram of the social preference assay. **(B)** No significant differences in investigation time of social and object contacts were observed between GH and jSI mice [2-way ANOVA, contact (social and object) x housing (GH and jSI) interaction F_(1. 40)_ = 3.050, p = 0.0884; contact F_(1. 40)_ = 14.76, p = 0.0004; housing F_(1. 40)_ = 0.00076, p = 0.9781, n = 11 (GH), n = 11 (jSI)]. **(C)** No significant difference in social preference was observed between GH and jSI mice [two-tailed *t*-test, t_20_ = 1.344, p = 0.1939, n = 11 (GH), n = 11 (jSI)]. **(D)** Schematic diagram of the social recognition assay. **(E)** Time spent in contact with a novel stranger by juvenile socially isolated (jSI) mice was significantly lower than that by group-housed (GH) mice [2-way ANOVA, contact (novelty and familiar) x housing (GH and jSI) interaction F_(1. 40)_ = 13.20, p = 0.0008; contact F_(1. 40)_ = 0.4613, p = 0.5009; housing F_(1. 40)_ = 0.1073, p = 0.7449; Tukey test p = 0.0376 (GH vs jSI in familiar), n = 11 (GH), n = 11 (jSI)]. **(F)** Social recognition in jSI mice was significantly lower than in GH mice [two-tailed *t*-test, t_20_ = 3.390, p = 0.0029, n = 11 (GH), n = 11 (jSI)]. GH, Group-Housed; jSI, Juvenile Social Isolation; S, Social; O, Object; N, Novelty; F, Familiar; ANOVA, Analysis of Variance. * 0.01 ≤ p < 0.05; ** 0.001 ≤ p < 0.01.

Numerous lines of evidence from human neuroimaging and rodent studies (34–38) have suggested that the evolutionarily conserved medial PFC is a component of a network governing social behavior. Therefore, we examined whether NARP-related functional alterations in the prefrontal excitatory and inhibitory circuitry in socially isolated mice could affect the social behavior of adult jSI mice. In a three-chamber social preference assay, jSI mice showed social interactions comparable to those of their GH littermates (**Figures 3A–C**, p = 0.19). During the social memory task, GH mice spent significantly more time exploring the novel stranger mice. However, jSI mice failed to differentiate between the novel stranger and familiar mice (**Figures 3D–F**, p = 0.0003). The behavioral deficits in adult jSI mice remained consistent throughout the entire 10-minute observation period (**Supplementary Figure 1**). This disparity in response to the novel stranger mouse cannot be attributed to motor or activity anomalies, as both GH and jSI mice exhibited similar locomotor activity during the first 10-minute phase in the three-chamber apparatus (**Supplementary Figure 2**, p = 0.36). These findings suggest that social isolation during the critical period influences certain aspects of social behavior in adult mice.

### 3.4 Lower expression of the NARP gene in ASD LCLs may be related to ASD pathophysiology

**Figure 4:**
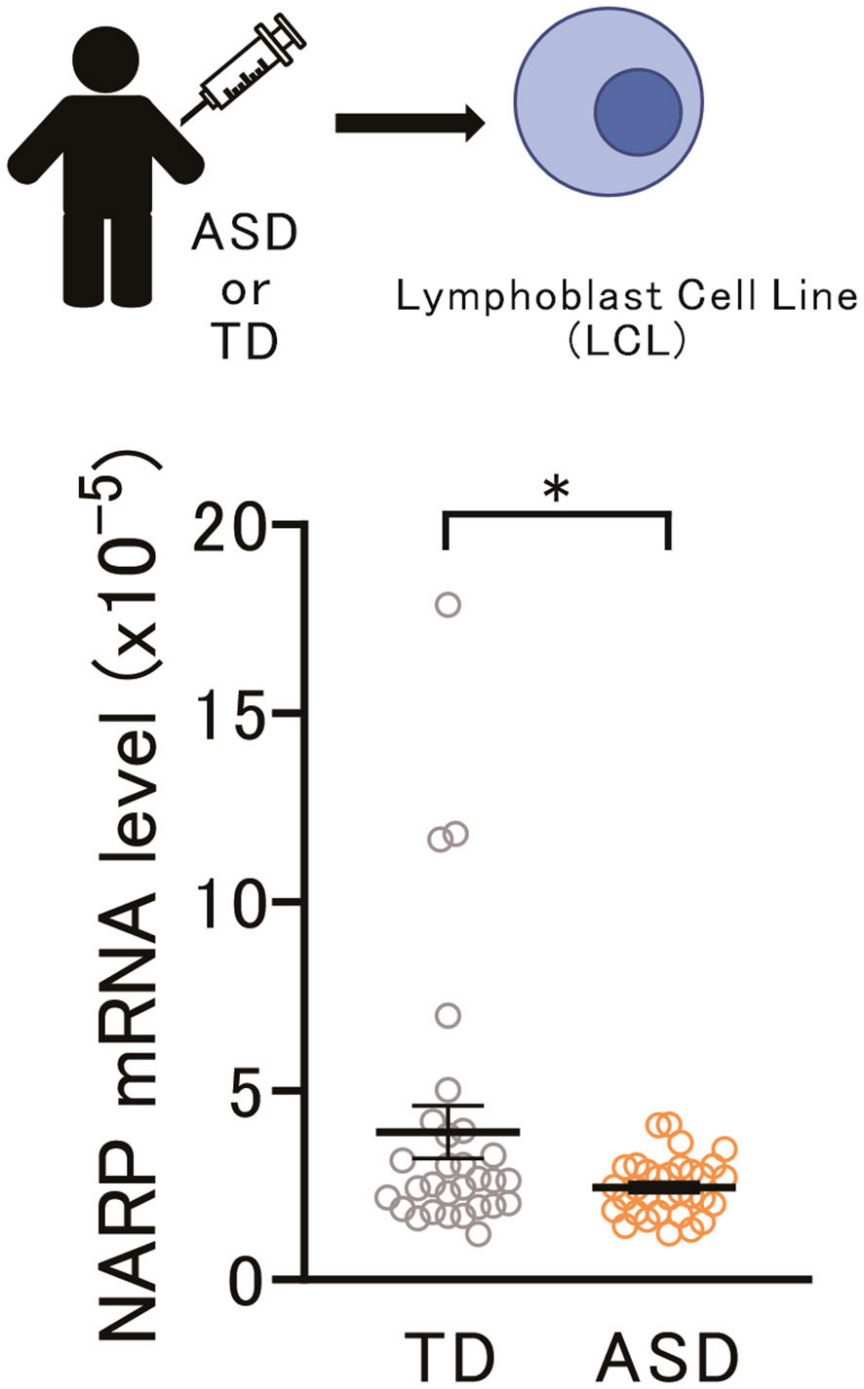
Lower NARP gene expression in ASD LCLs may relate to ASD pathophysiology. Top: Schematic diagram of NARP mRNA assay in LCLs from adolescents with Typically Developing (TD) individuals and those with ASD. Bottom: NARP mRNA expression was lower in ASD LCLs than in TD LCLs [two-tailed *t*-test, t_59_ = 2.172, p = 0.0339, n = 29 (TD), n = 32 (ASD)]. NARP, Neuronal Activity-Regulated Pentraxin; ASD, Autism Spectrum Disorder; LCLs, Lymphoblastoid Cell Lines; TD, Typically Developing. * 0.01 ≤ p < 0.05.

These data underscore the significant involvement of the NARP gene in the neuronal and behavioral aspects of socially isolated mice. Obtaining neurons or neuronal cells from living human subjects is challenging. However, previous studies have suggested that LCLs can be used to identify biologically plausible links between candidate genes and various psychiatric disorders (39–41), including ASD (42). Therefore, we assessed the NARP mRNA levels in LCLs derived from adolescents with ASD and typically developing (TD) adolescents. As shown in **Figure 4**, a marked decrease in NARP expression in individuals with ASD compared to TD individuals (p = 0.047) was identified. Since social isolation is considered a critical factor in the development or exacerbation of ASD and is common among patients with ASD, these data suggest that alterations in NARP expression may contribute to the pathophysiology of ASD.

## 4 Discussion

In the present study, using a comprehensive approach that integrates electrophysiological, gene and protein expression, and behavioral analyses, we investigated the timing and molecular mechanisms underlying the changes in prefrontal excitatory and inhibitory circuitry in socially isolated mice. After juvenile social isolation at P35, we found that it immediately affected the excitability of medial PFC layer 3 pyramidal cells, potentially contributing to subsequent dysfunction in the adult medial PFC. Furthermore, the altered expression of NARP, an immediate early gene product, appeared to influence the expression of PV neurons in socially isolated mice, which might affect the electrophysiological properties of prefrontal excitatory and inhibitory neurons in adult mice. We also observed that while socially isolated mice exhibited similar levels of social interaction as control mice, they had difficulty distinguishing between novel and familiar mice during the social memory task, indicating that social isolation during the critical period affects certain aspects of social behavior in adult mice. Finally, given the social behavioral deficits in individuals with ASD, decreased NARP expression in ASD LCLs might indicate its potential involvement in ASD pathophysiology. Collectively, we suggest the importance of the role of the NARP gene in the neuronal and behavioral features of socially isolated mice and its potential association with psychiatric disorders, such as ASD.

Our electrophysiological study revealed that social isolation during the critical period can immediately lead to changes in the firing properties of pyramidal neurons in layers 3 and 5 of the medial PFC, which in turn may affect the functional properties and communication within the cortical circuitry. Previous studies have shown that layer 3 pyramidal neurons, which are generally smaller than layer 5 pyramidal neurons (43), are involved in the integration and processing of information within the medial PFC. These neurons primarily project to other cortical areas, including the contralateral medial PFC and other association cortices, and thus play a crucial role in intracortical communication (44). In contrast, layer 5 pyramidal neurons, with their distinct electrophysiological properties, contribute to the output of the medial PFC to subcortical structures (45). This diverse innervation pattern allows layer 5 neurons to modulate various behavioral and cognitive processes. Furthermore, the reciprocal connections between layers 3 and 5 neurons play a crucial role in the encoding, integration, and processing of information within the medial PFC (46). Alterations in the intrinsic properties of neurons in these layers due to social isolation during the critical period may disrupt the functional balance and communication between these layers. Collectively, this disruption might impair the overall functionality of the medial PFC, which is essential for higher-level cognitive processes such as working memory and social behavior. However, our current study does not directly track the long-term persistence of these electrophysiological alterations. Several lines of evidence suggest that social isolation during a critical period could have long-lasting effects on several behavioral deficits (47, 48) and electrophysiological changes, particularly in layer 5 pyramidal neurons (6, 8, 49, 50). Therefore, further longitudinal studies are necessary to explicitly trace the electrophysiological changes in layer 3 pyramidal neurons from the juvenile period into adulthood.

Altered expression of NARP, an immediate early gene product that appears to influence the expression of PV in socially isolated mice at P35 was observed. This alteration may be associated with the electrophysiological properties of prefrontal excitatory and inhibitory neurons in socially isolated adult mice. NARP is an activity-dependent gene product that is predominantly secreted by excitatory pyramidal neurons, such as those found in layers 3 and 5 and is involved in the development of excitatory synapses exclusively in PV neurons (23). The significant effect of housing conditions on NARP mRNA expression suggests that social isolation during the critical period may disrupt the normal activity-dependent regulation of NARP expression in these pyramidal neurons. This disruption could potentially lead to altered development of excitatory synapses in PV neurons, which in turn might affect the balance between excitation and inhibition in the cortical circuitry during critical periods of developmental plasticity (32, 51). Furthermore, given the role of PV neurons in regulating the activity of excitatory pyramidal neurons, including those in layers 3 and 5, decreased PV expression in jSI mice may result in an imbalance between excitation and inhibition within the prefrontal circuitry, potentially contributing to medial PFC dysfunction during adulthood. However, the present study revealed that NARP and PV mRNA expression in the medial PFC of jSI exhibited distinctive patterns when compared to the GH. Although NARP has been implicated as a crucial regulator of excitatory and inhibitory synaptic scaling, particularly in PV neurons (23), the lack of a direct correlation between NARP and PV expression in our study suggests that additional molecular factors may be involved in regulating the balance between excitation and inhibition in the medial PFC of socially isolated mice. Alternatively, recent studies have identified various subtypes of inhibitory interneurons, including PV neurons, based on their morphoelectric and transcriptomic profiles (52, 53). Considering our previous finding that electrophysiological functions of a specific PV neuron subtype were immediately altered following social isolation at P35 (6), the observed PV expression levels at P35 might partially reflect a composite effect involving multiple PV neuron subtypes. Therefore, further investigations are required to elucidate the underlying molecular mechanisms and environmental factors that contribute to the observed changes in the excitatory and inhibitory circuits in the medial PFC of socially isolated mice.

In this study, we found that social isolation during the critical period affects certain aspects of social behavior in adult mice, specifically their ability to distinguish between novel and familiar mice during social memory tasks. Previous studies have demonstrated that the medial PFC plays a crucial role in social memory processing (54, 55). Therefore, the electrophysiological alterations observed in our study, particularly in medial PFC pyramidal neurons, could potentially contribute to the social memory deficits observed in socially isolated mice. However, research on the effects of social isolation during critical periods has produced mixed results regarding social behavior, including both reduced and intact social interaction (56–58). In our study, socially isolated mice exhibited comparable levels of social interaction to control mice. Given that perturbations in synaptic levels, such as spine density or gene expression, might also yield varied findings (48, 59), this discrepancy might be attributed to differences in experimental design, housing conditions, or the specific age at which isolation occurred. We suggest that the effects of social isolation on social behavior are complex and may vary depending on the framework or paradigm of social behavior examined. It would be interesting to investigate whether the mode of re-socialization, as studied by Makinodan et al. (58), influences the electrophysiological and molecular changes observed in socially isolated mice.

Given that excitatory and inhibitory synaptic dysfunction is considered a shared pathophysiological mechanism in psychiatric disorders, including ASD (60, 61), the significant reduction in NARP expression in LCLs from adolescents with ASD supports the hypothesis that NARP may play a role in the development or exacerbation of ASD symptoms. Although LCLs are not neuronal cells, several studies have demonstrated altered gene expression profiles in LCLs derived from individuals with ASD compared to TD (62–65). These findings suggest that LCLs may serve as a useful model for investigating molecular changes associated with ASD. Further investigation is required to confirm the role of NARP in the pathophysiology of ASD and to explore whether modulating NARP expression or function could be a potential therapeutic target for treating ASD and related conditions.

Our study has a few limitations. Firstly, our analysis focused solely on the medial PFC, an important brain region in the regulation of social behaviors (34); however, it is not the only brain region involved in these processes. Other regions, such as the amygdala (66), hippocampus (67, 68), and other cortical areas (69, 70), also play crucial roles in social behavior and cognition. Future studies employing optogenetic and chemogenetic approaches could explore the effects of social isolation on these other brain regions for a more comprehensive understanding of its impact on the brain. Secondly, we did not examine molecular changes at the cellular or laminar resolutions within the medial PFC. Investigating the effects of social isolation on specific cell types, such as different subpopulations of pyramidal neurons or interneurons, or the expression of molecular markers in distinct cortical layers, could provide further insights into the underlying mechanisms of the observed alterations in excitatory and inhibitory circuitry. Finally, although our study revealed significant alterations in NARP expression in LCLs generated from individuals with ASD, it is important to note that LCLs are not neuronal cells, and thus may not fully recapitulate the molecular and cellular processes occurring in the brain. Furthermore, the absence of clinical indices of sociability in our dataset precludes a direct correlation between NARP mRNA expression and sociability scores in ASD, which would have provided deeper insights into the biological underpinnings of social behavior in ASD. Future studies should incorporate these measures to elucidate the role of NARP in individuals with ASD. However, the significant reduction in NARP expression in ASD LCLs, combined with our findings of altered NARP and PV expression, electrophysiological properties, and social behavior in socially isolated mice, offers valuable insights into its role in the pathophysiology of ASD.

In conclusion, the present study provides novel insights into the impact of juvenile social isolation on the excitability of medial PFC pyramidal cells and expression of NARP, with subsequent effects on parvalbumin inhibitory neurons. Furthermore, we suggest a potential link between NARP expression, social behavior, and ASD pathophysiology. A deeper understanding of the molecular mechanisms underlying the effects of social experiences during critical periods may inform the development of early intervention strategies to mitigate the long-term consequences of aberrant juvenile social interactions on brain development and function.

## Data availability statement

The raw data supporting the conclusions of article will be made available by the authors, without undue reservation.

## Ethics statement

The studies involving humans were approved by the Nara Medical University Ethics Committee. The studies were conducted in accordance with the local legislation and institutional requirements. Written informed consent for participation in this study was provided by the participants’ legal guardians/next of kin. The animal study was approved by the Animal Care and Use Committee of Nara Medical University. The study was conducted in accordance with the local legislation and institutional requirements.

## Author contributions

YY: Formal analysis, Investigation, Writing – original draft, Writing – review & editing, Data curation. KOka: Formal analysis, Funding acquisition, Investigation, Writing – original draft, Writing – review & editing, Data curation. KY: Writing – review & editing, Investigation. KOku: Writing – review & editing, Investigation. TKo: Writing – review & editing, Investigation. MT: Writing – review & editing, Investigation, Resources. RT: Writing – review & editing, Investigation. YN: Writing – review & editing, Investigation. DI: Writing – review & editing, Investigation. TY: Writing – review & editing, Investigation. MM: Writing – review & editing, Investigation, Supervision. HY: Writing – review & editing, Investigation, Supervision. YS: Writing – review & editing, Supervision. HM: Writing – review & editing, Resources, Supervision. TKi: Supervision, Writing – review & editing. SK: Formal analysis, Funding acquisition, Investigation, Methodology, Supervision, Writing – original draft, Writing – review & editing, Data curation.
