# Supplementary material for: NARP-related alterations in the excitatory and inhibitory circuitry of socially isolated mice: developmental insights and implications for autism spectrum disorder
Source: Front Psychiatry. 2024 Jun 6;15:1403476. doi: 10.3389/fpsyt.2024.1403476 (PMC11187327; doi:10.3389/fpsyt.2024.1403476)
Supplement: Supplementary file 1 [file Image_1.pdf]

Supplementary Material

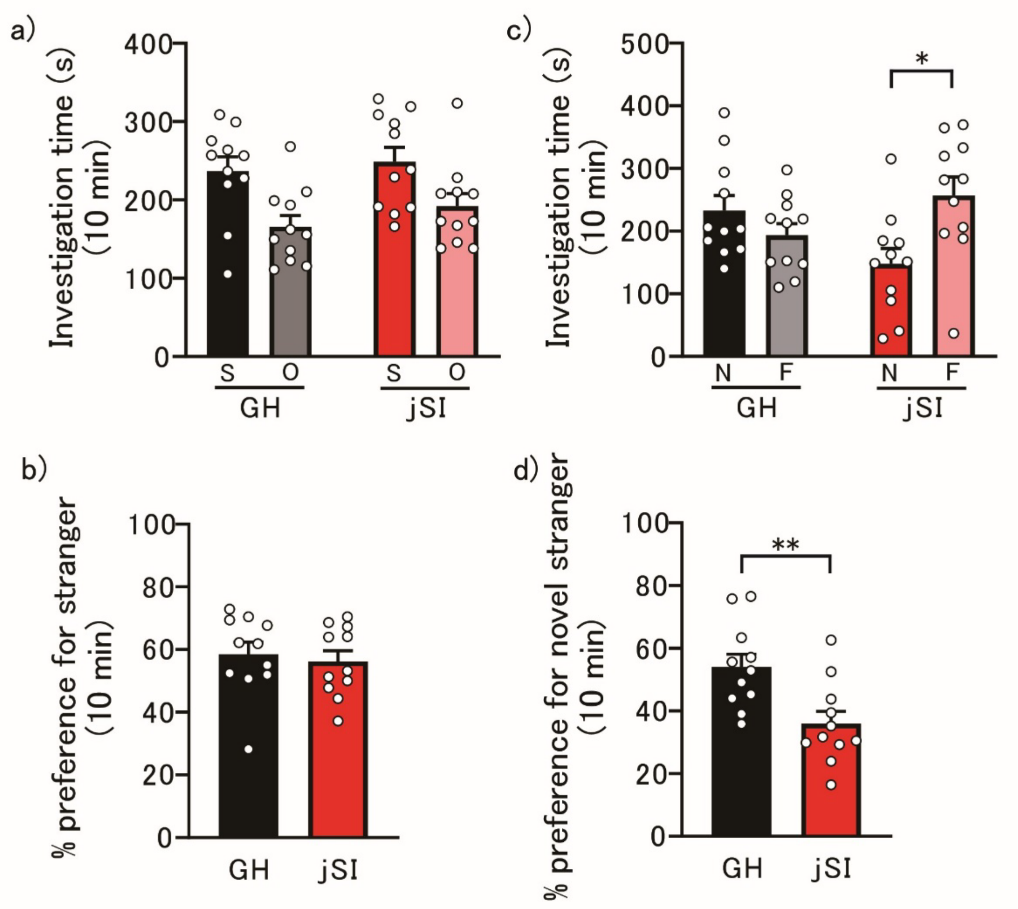

Supplementary Figure 1. Analysis of the three-chamber test monitoring 10-minute observation

a) No significant differences in investigation time of social and object contacts were observed between GH and jSI mice (2-way ANOVA, contact (social and object) x housing (GH and jSI) interaction; $F_{(1. 40)} = 0.1800$ $p = 0.6736$; contact $F_{(1. 40)} = 14.31$ $p = 0.0005$; housing $F_{(1. 40)} = 1.285$ $p = 0.2638$, n = 11 (GH), n = 11 (jSI)). b) No significant difference in social preference was observed between GH and jSI mice (two-tailed *t*-test, $t_{20} = 0.4385$, $p = 0.6657$, n = 11 (GH), n = 11 (jSI)). c) Time in contact to novel stranger of jSI mice was significantly lower than that in GH mice (2-way ANOVA, contact (novelty and familiar) x housing (GH and jSI) interaction $F_{(1. 40)} = 9.256$ $p = 0.0041$; contact $F_{(1. 40)} = 2.074$ $p = 0.1576$; housing $F_{(1. 40)} = 0.1989$ $p = 0.6580$; Tukey test $p = 0.0149$ (jSI in Novelty vs jSI in Familiar), n = 11 (GH), n = 11 (jSI)). d) social recognition in jSI mice was significantly lower than in GH mice (two-tailed *t*-test, $t_{20} = 3.189$, $p = 0.0046$, n = 11 (GH), n = 11 (jSI)).

Abbreviations: GH: Group-Housed; jSI: Juvenile Social Isolation; S: Social; O: Object; N: Novelty; F: Familiar; ANOVA: Analysis of Variance

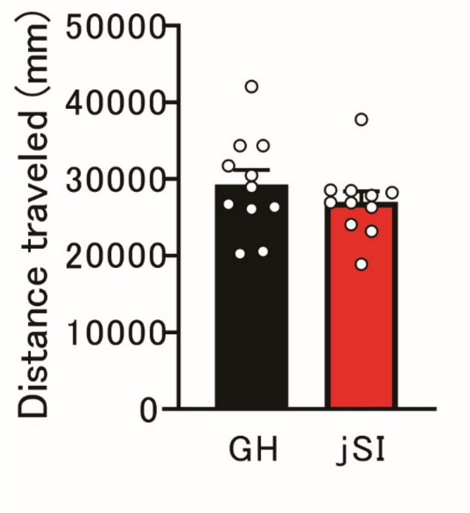

Supplementary Figure 2. Juvenile social isolation does not affect locomotive behavior.

There was no significant difference in locomotion between the GH and jSI mice (2-tailed *t*-test, t_{20} = 0.9473, p = 0.3548, n = 11 (GH), n = 11 (jSI)).
