# Supplementary material for: NARP-related alterations in the excitatory and inhibitory circuitry of socially isolated mice: developmental insights and implications for autism spectrum disorder
Source: Front Psychiatry. 2024 Jun 6;15:1403476. doi: 10.3389/fpsyt.2024.1403476 (PMC11187327; doi:10.3389/fpsyt.2024.1403476)
Supplement: Supplementary file 2 [file Table_1.docx]

***Supplementary Material***

|  | TD | ASD |
| --- | --- | --- |
| Sample number | 29 | 32 |
| Male : Female | 15 : 14 | 22 : 10 |
| Age (Mean ± SD) | 12.0 ± 2.3 | 11.7 ± 2.7 |

TD: typically developing (TD) humans, ASD: autism spectrum disorder

Supplementary Table 1. Demographic characteristics of subjects used by lymphoblastoid cell line study

| Gene | Species | Accession # | Position | Forward Primer (F)  Reverse Primer (R) |
| --- | --- | --- | --- | --- |
| ACTB^1^ | Mus  musculus | NM_007393 | 955-1049 | (F) CCTCTATGCCAACACAGTGC  (R) TGCTAGGAGCCAGAGCAGTA |
| Cyclo^2^ | Mus  musculus | NM_008907 | 383-445 | (F) CTGCACTGCCAAGACTGAAT  (R) CCTTCTTTCACCTTCCCAAA |
| GAPDH^3^ | Mus  musculus | NM_008084 | 431-494 | (F) CATGTTTGTGATGGGTGTGA  (R) TGCATTGCTGACAATCTTGA |
| NARP | Mus  musculus | NM_016789 | 1265-1354 | (F) CCACGCAGGCCTTTGTTG  (R) AGCAGTTGGCCATGTTGA |
| PV^4^ | Mus  musculus | NM_013645 | 87-193 | (F) CAGACTCCTTCGACCACAAA  (R) GCCACTTTTGTCTTTGTCCA |
| ACTB^1^ | Human | NM_001101 | 1146-1246 | (F) GATGTGGATCAGCAAGCA  (R) AGAAAGGGTGTAACGCAACTA |
| Cyclo^2^ | Human | NM_021130 | 159-284 | (F) GCAGACAAGGTCCCAAAG  (R) GAAGTCACCACCCTGACAAC |
| GAPDH^3^ | Human | NM_002046 | 556-642 | (F) TGCACCACCAACTGCTTAGC  (R) GGCATGGACTGTGGTCATGAG |
| NARP | Human | NM_002523 | 1271-1360 | (F) CCACTCAGGCATTTGTCG  (R) AGCAGTTGGCGATGTTGA |

1. β-actin, 2. cyclophilin, 3. glyceraldehyde-3-phosphate dehydrogenase, 4 parvalbumin

Supplementary Table 2. Primer Design used in this study
